# PROFASA—a web-based protein fragment and structure analysis workstation

**DOI:** 10.3389/fbioe.2023.1192094

**Published:** 2023-07-21

**Authors:** Yanlin Mi, Stefan-Bogdan Marcu, Sabin Tabirca, Venkata V. B. Yallapragada

**Affiliations:** ^1^ School of Computer Science and Information Technology, University College Cork, Cork, Ireland; ^2^ SFI Centre for Research Training in Artificial Intelligence, University College Cork, Cork, Ireland; ^3^ Faculty of Mathematics and Informatics, Transylvania University of Brasov, Brasov, Romania; ^4^ Centre for Advanced Photonics and Process Analytics, Munster Technological University, Cork, Ireland; ^5^ Tyndall National Institute, Cork, Ireland

**Keywords:** protein modeling, computational biology, proteins, edutainment, gamification, molecular visualisation, bioinformatics

## Abstract

**Introduction:** In the field of bioinformatics and computational biology, protein structure modelling and analysis is a crucial aspect. However, most existing tools require a high degree of technical expertise and lack a user-friendly interface. To address this problem, we developed a protein workstation called PROFASA.

**Methods:** PROFASA is an innovative protein workstation that combines state-of-the-art protein structure visualisation techniques with cutting-edge tools and algorithms for protein analysis. Our goal is to provide users with a comprehensive platform for all protein sequence and structure analyses. PROFASA is designed with the idea of simplifying complex protein analysis workflows into one-click operations, while providing powerful customisation options to meet the needs of professional users.

**Results:** PROFASA provides a one-stop solution that enables users to perform protein structure evaluation, parametric analysis and protein visualisation. Users can use I-TASSER or AlphaFold2 to construct protein models with one click, generate new protein sequences, models, and calculate protein parameters. In addition, PROFASA offers features such as real-time collaboration, note sharing, and shared projects, making it an ideal tool for researchers and teaching professionals.

**Discussion:** PROFASA’s innovation lies in its user-friendly interface and one-stop solution. It not only lowers the barrier to entry for protein computation, analysis and visualisation tools, but also opens up new possibilities for protein research and education. We expect PROFASA to advance the study of protein design and engineering and open up new research areas.

## Introduction

### Myriad of computational tools for tools

Understanding protein structure and function is a crucial aspect of life sciences. Commercially, synthetic proteins are poised to drive a major sector of synthetic biology (Walker et al, 2021). Protein engineering market is expected to reach USD 3,023.29 million by 2027^1^. The idea of using computational tools to enhance our scientific arsenal to study proteins is well documented (Walker et al, 2021). Highly significant and difficult problems (Kuhlman and Bradley, 2019) such as predicting the 3D structure of a protein from its amino acid sequence are now being solved with great accuracy by tools such as AlphaFold2, using advanced deep learning models. Entirely novel structures that never existed in nature can now be generated using *de novo* protein design (Gront et al, 2011). During COVID-19 pandemic, 3D protein visualisation is employed in the teaching of undergraduate medicinal chemistry courses to investigate drug-target interactions (“Distant learning challenges and solutions,” 2020).

Protein engineering will become increasingly more efficient and precise in the future as artificial intelligence and deep learning progress. When it comes to protein structure prediction, we’re living in exciting times, AlphaFold2 by Google’s Deepmind has already been a blockbuster for the prediction of protein structures with high accuracy. Improvements are also expected in areas such as better Deep Learning-based (DL-Based) algorithms for Multiple Sequence Alignments (MSA) generation; transformer-based approaches for protein structure prediction; DL-Based approaches for multi-domain protein structure prediction and so on (Pakhrin et al, 2021). However, as in other fields, one obstacle to the wider use of deep learning in protein structure informatics is the black-box nature of deep learning models. In this context, the development of Explainable Artificial Intelligence (XAI) approaches to improve the interpretability of protein structure predictions is an emerging trend in the area. Such as InterPretContactMap which was developed by Cheng’s Lab (Adhikari et al, 2018). It uses two attention mechanisms (sequence and regional) in the Convolutional Neural Network (CNN) framework to do contact map prediction and improves the contact map prediction results as well as provides some level of interpretability, providing some insights into the key fold-determining residues in the protein. Therefore, it is important to use advances in deep learning algorithms to fill the existing gap between protein sequence to protein structure, and XAI might become one of the methods to achieve the goal (Pakhrin et al, 2021). With *de novo* protein design methods, as the protein structure database expands, protein structures with novel functions can be generated through AI. All these necessitate a lot of mathematical power, thus, as with structure prediction, deep learning might propel the study of protein design and engineering to new heights. There are many freely available protein tools on the market. For sequence alignment, BLAST has long been a widely used tool (Altschul et al, 1997). For structure prediction, Rosetta and I-TASSER are the most popular online tools to process protein structure analysis (Yang and Zhang, 2015; Leman et al, 2020), and the recent revolution AlphaFold2 is based on deep learning and millions of structures have already been generated by it (Cramer, 2021). Apart from structure prediction, advancements can also be seen in protein visualisation and edutainment. Tools such as Chimera, Rasmol, Pymol and Csynth offer excellent features to highlight and visualise molecular structures in great detail (Yuan et al, 2017; Pettersen et al, 2021; Todd et al, 2021; Fraley et al, 2022). Recently, efforts have also been made to provide immersive viewing with virtual reality and augmented reality (Yallapragada et al, 2021). In edutainment, Fold it by Rosetta is a fantastic resource for understanding the 3D mechanics of protein structures in a gamified environment (Curtis, 2015). The above mentioned tools are only a snapshot of the rapidly expanding field of protein design. Although there has been extraordinary progress in the individual tools, a unified interface that can act as a one-stop-shop for all protein needs is required to bring the power of protein design to en masses and would expand the horizons of protein design.

### One tool to rule them all

**FIGURE 1:**
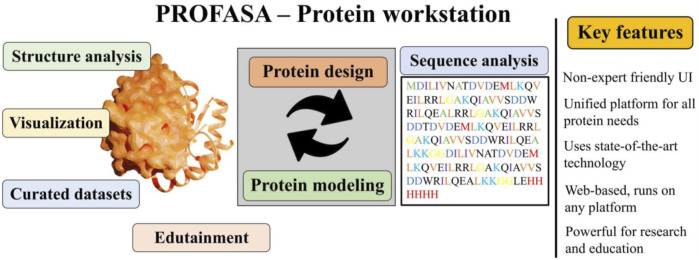
PROFASA, combines all the major computational tools and provides a single platform interface for computational protein works.

PROFASA (Figure 1: *PROFASA*) is an *in silico* workstation for protein structure evaluation, parametric analysis, and protein visualisation. It provides i) a unified interface for rapid conserved domain analysis of input sequences and displays the results using a molecular visualisation UI ii) users can construct protein models with one click using the I-TASSER or the AlphaFold2, iii) it can fold numerous input sequences, create new protein sequences, models, and calculate protein parameters, iv) it uses tables and plots to display and compare all data, these results provide a guide to the expected protein performance prediction using Function2Form (F2F) plot (Yallapragada et al, 2020).

**FIGURE 2:**
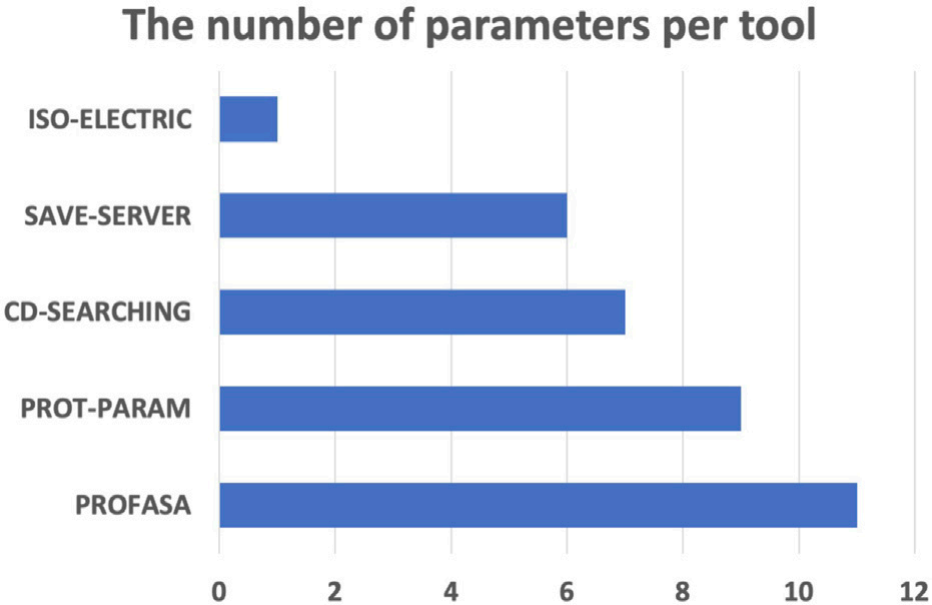
The number of parameters per tool.

**FIGURE 3:**
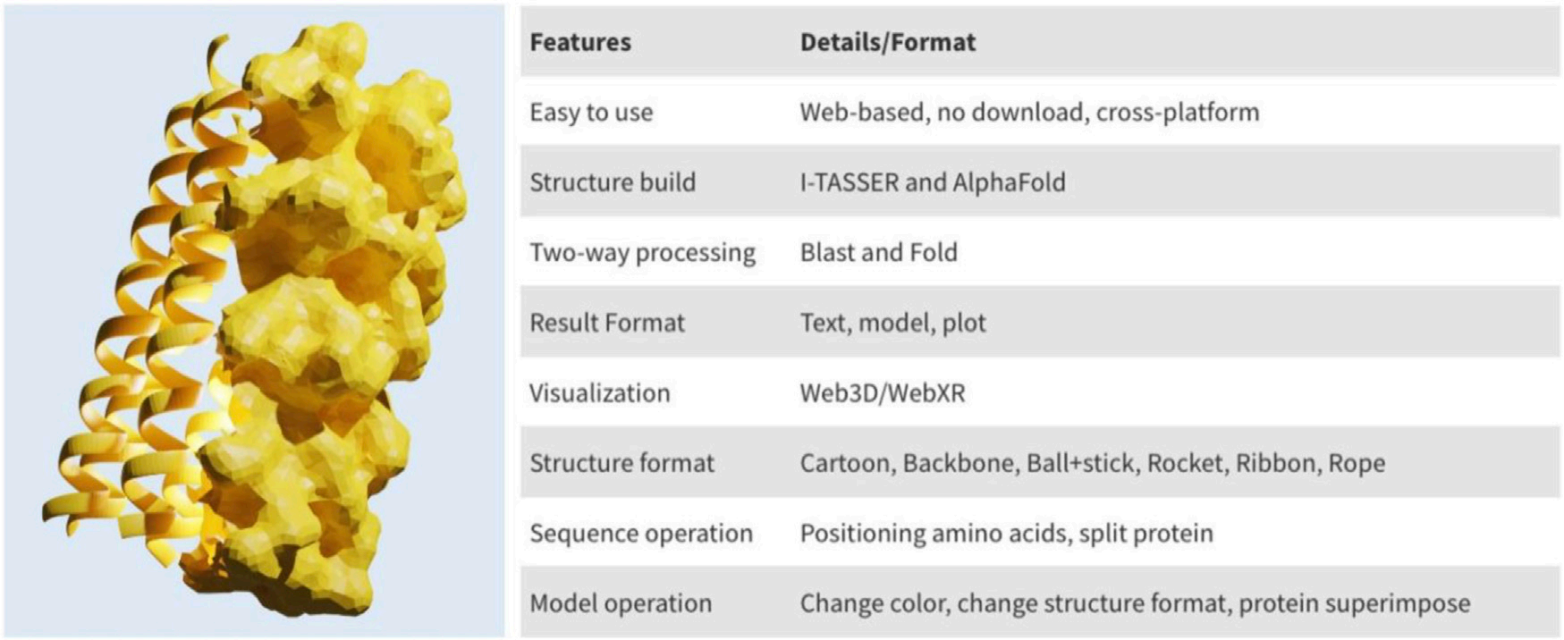
PROFASA key features.

PROFASA has fast model generation and structural analysis time and is faster than most tools on the market, as shown in the (Table 1: *Model generation timetable*). Users only have to submit a sequence to get parameters like accessibility, instability, hydrophobicity, isoelectricity, size, and Ramachadran plot score (Figure 2: *The number of parameters per tool*). Drawing upon the precision of well-established bioinformatics tools, PROFASA provides accurate results across all its features. Its sequence alignment functionality uses the NCBI BLAST toolkit, which is known for its reliable sequence comparison capabilities. Structure prediction in PROFASA is handled through I-TASSER^2^ and AlphaFold2^2^, both recognized as leaders in protein structure prediction, thus the accuracy in this aspect is assured. Furthermore, calculations pertaining to protein superimposition and other protein parameters are performed based on standard, scientifically accepted formulas (see Materials and Methods section), ensuring the results generated are as precise as these foundational formulas. PROFASA has an excellent user interface, provides an interactive experience, and is simple to operate. When compared to most existing protein analysis tools, it is appropriate for both professionals and non-professionals due to its ease of use and data visualisation. This is a key feature in reducing the entry barrier for protein computation, analysis, and visualisation tools, allowing more people to participate in protein research and education (Figure 3: *PROFASA key features*).

**TABLE 1:** Model generation timetable

Uniprot ID	Length	Execution Duration			
		PROFASA with AlphaFold2 (with 6 parameters calculation) (min)	PROFASA with I-TASSER (with 6 parameters calculation)	I-TASSER Server (with 1 parameter calculation)	AlphaFold2 Colab (with 1 parameter calculation) (min)
P63212	57	38	9h52min	12h32min	10
P09210	100	40	14h13min	21h05min	14
P61626	128	40	13h13min	21h51min	13
O75469	135	44	16h34min	18h34min	15

## Materials and methods

### Architecture design

**FIGURE 4:**
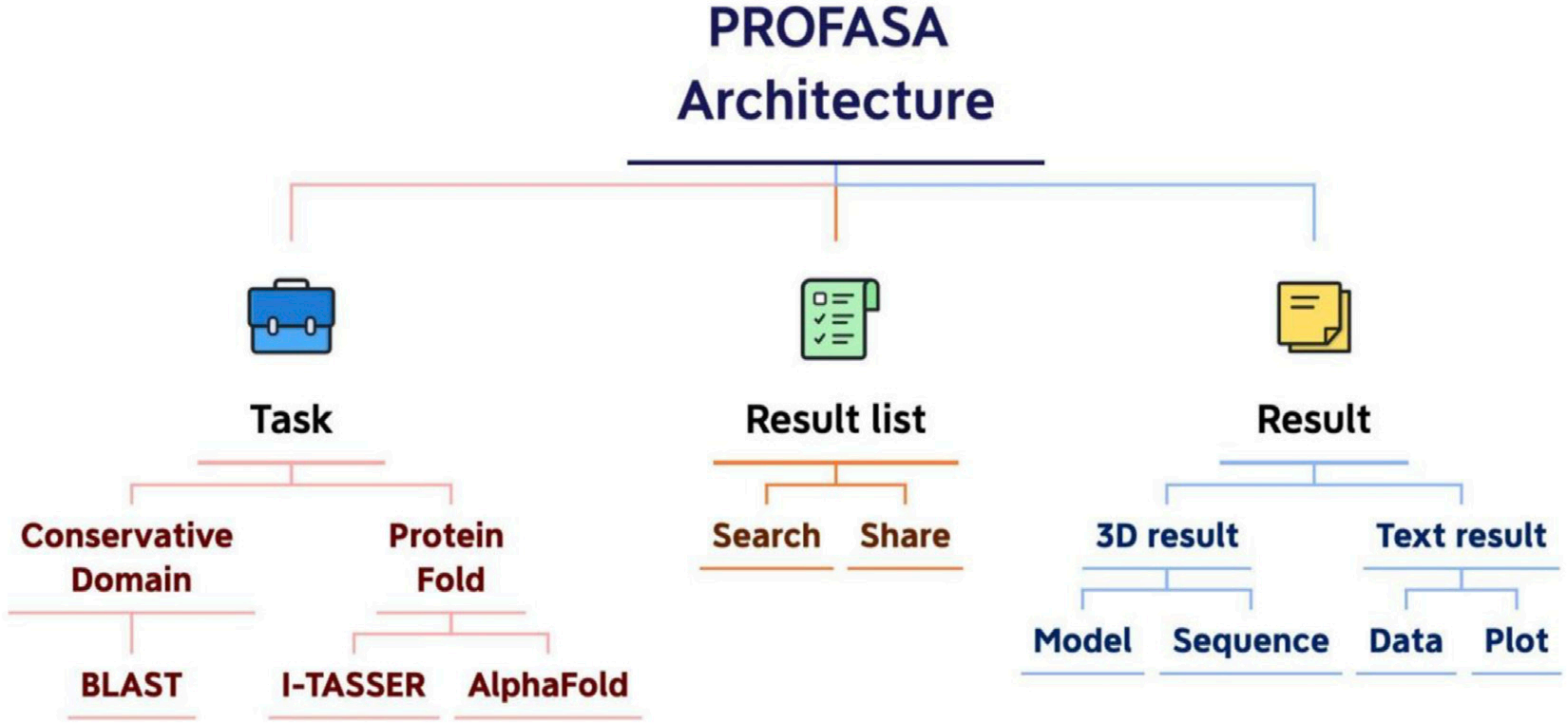
PROFASA architecture.

Serving as a one-stop workstation, we aim to provide the users with a platform for all the protein sequence and structure based analysis. The architecture design specifies the goal, key functions, activity elements, and important processes, as well as the nature of their interconnection (Gharajedaghi, 2011). Each module of the PROFASA project can be split down based on the user requirements as shown in (Figure 4: *PROFASA architecture*)

The application architecture of PROFASA describes design and development patterns and techniques. When developing applications, a strong application architecture gives a roadmap and recommended practices to follow (Eder and Missikoff, 2003). Applications in PROFASA are divided into presentation layer, logical layer, and computing layer. The front end is the display layer, which contains the Home page, Blast page, Fold page, Resources page, Result List page, Text-result page and 3D-result page. Node.js part of the backend is a logical layer, which is divided into account service, mail service, note service, sequence service, I-TASSER service, AlphaFold service, and parameter service. Finally, the command line program part is the computing layer, which is divides into RpsBlast, I-TASSER, AlphaFold2 and Ramachandran.

### Front end design

PROFASA uses TypeScript as its primary programming language because it operates in a web environment. TypeScript is a JavaScript superset that adds types, interfaces, and other useful features to JavaScript (Bierman et al, 2014). ReactJS, the most popular framework in the world, was chosen for the technical decision of the programming development framework. It’s a JavaScript library for designing user interfaces using JSX quickly and effortlessly (TSX is used in PROFASA). UmiJS is used to handle the react-router and react-state in this project. It is an enterprise-level React application framework created by Alibaba that includes comprehensive routing functions (basically an improved encapsulation of react-Router and react-router-DOM) and a set of state management techniques based on fixed hooks. This is a lot easier than Redux. PROFASA is a powerful and versatile work station with a wide range of complicated and comprehensive capabilities. It uses two high-quality JavaScript libraries: Three.js and ngl.js, to perform its main duties of visual rendering and interaction with protein 3D models. Ricardo Cabello, aka Mr.Doob, designed Three.js in 2010 (Danchilla, 2012). It has a variety of draw modes and can fall back to a 2D rendering environment if WebGL isn’t available. The amount of initial effort or “boilerplate” required is reduced with default settings (Danchilla, 2012). The lighting, camera, camera controller, renderer, animation, and mouse interaction events in the scene are all built using Three.js. Because users may transition between multi-model and single-model scenes with a single click, PROFASA needs to provide varied mouse hover and click interactions in different scenes, the 3D scenes feature particularly complicated mouse interactions. The ngl.js is used to load and parse PDB files, which are available objects in Three.js. In addition, one of PROFASA’s most important functions is the ability to superimpose, extract protein sequences and change the color and shape of selected fragments, which is also dependent on ngl.js. Multiple PDB files are loaded using ngl.js when users visit the 3D result page. Following successful loading, different colored 3D models will be rendered based on the quantity of models. When users change the style of the selected piece of the model, or the style of the entire model, it effectively destroys the old model and produces a new one using ngl.js. The ngl.js is also used by protein superimpose to determine comparable amino acids in two proteins and recalculate the locations of the proteins to finish the superimpose. The Root-mean-square Deviation (RMSD) is an essential statistic for determining the excellent and bad superimpose criterion for protein. The average distance between the atoms of the two stacked proteins is measured by the RMSD (Patel et al, 2019). The smaller the value, the closer the two overlaid proteins are in terms of exterior features and functions, and the more they converge. RMSD can be calculated using Formula 1. One of the first steps in calculating RMSD is to align the sequences. Sequence alignment is a method for matching protein sequences in order to find functional or structural similarities or differences between them (Chao et al, 2022). PROFASA calculates it using a matrix technique (Wang et al, 2017). Sequence alignment determines which residues in two sequences are identical, then locates the alpha carbon atoms that correspond to these residues and obtains their position coordinates using the protein object, which is generated when parsing the PDB file and will update the position coordinates of the atoms in it after superimpose of the protein. Then, after having all of the atoms’ location coordinates, Formula 2 could be used to get the RMSD. In addition, through the coordinates of these alpha carbon atoms, The Global Distance Test—Total Score (GDT_TS) can be calculated. When the two structures differ in detail, GDT_TS^3^,^4^ is better than RMSD^3^,^4^ in detecting superimpose similarity (Zemla, 2003). GDT_TS can be calculated by Formula 2, where GDT_PN denotes percent of residues under distance cutoff <= NÅ, as in Formula 3 shown.

$$RMSD\left(v,w\right)=\sqrt{\frac{\sum_{i=1}^{n}\left({\left(v_{ix}-w_{ix}\right)}^{2}+{\left(v_{iy}-w_{iy}\right)}^{2}+{\left(v_{iz}-w_{iz}\right)}^{2}\right)}{n}}$$ (1)

$$GDT\_TS=\frac{GDT\_P1+GPT\_P2+GPT\_P4+GPT\_P8}{4}$$ (2)

$$GDT\_PN=\frac{N\left[{\left(v_{x}-w_{x}\right)}^{2}+{\left(v_{y}-w_{y}\right)}^{2}+{\left(v_{z}-w_{z}\right.}^{2}\right]}{n}\times 100$$ (3)

**FIGURE 5:**
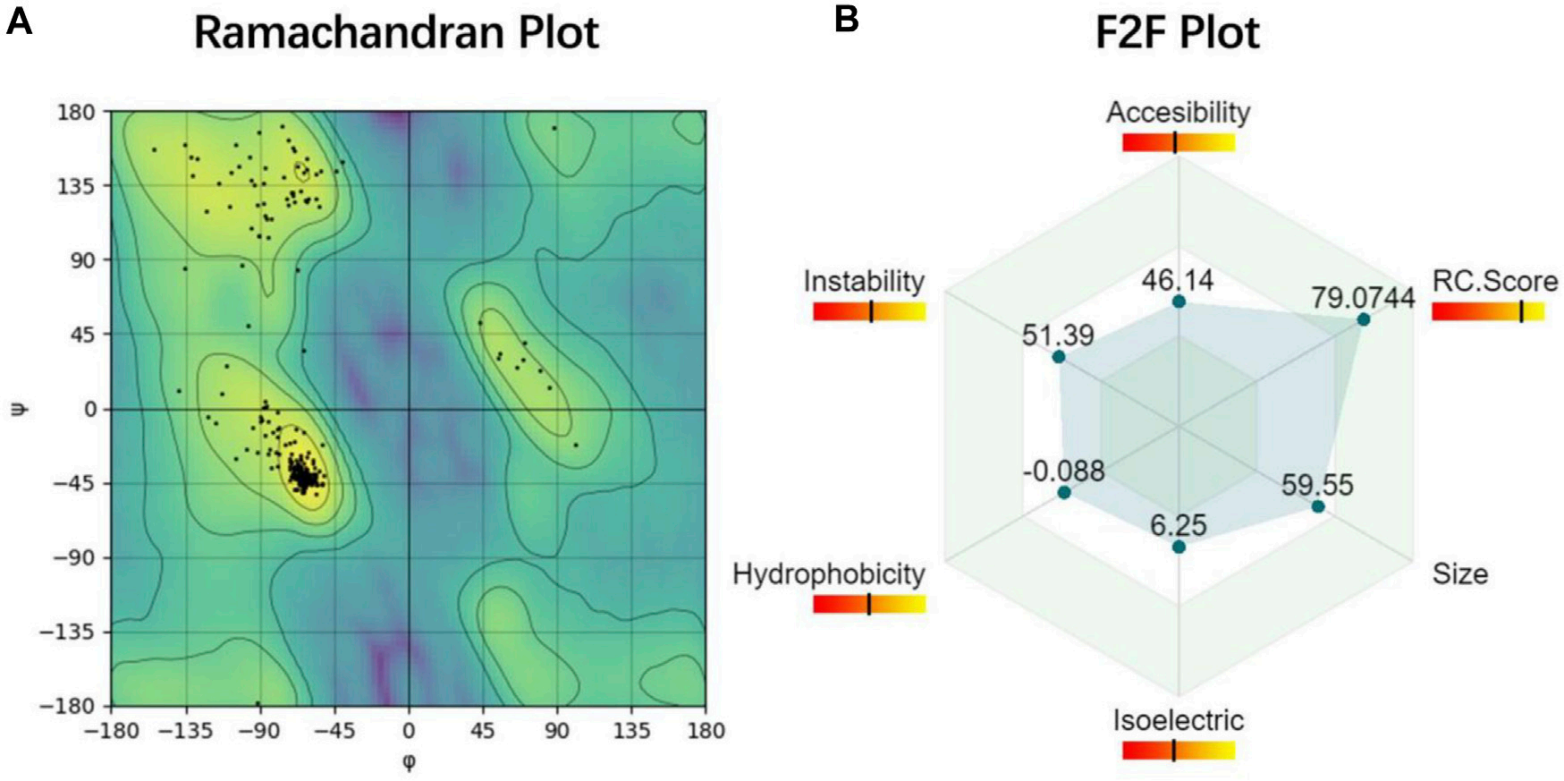
Ramachandran Plot and F2F Plot **(A)** Ramachandran plot is used to describe whether the conformation of the protein is reasonable (Hooft et al, 1997). **(B)** Function2Form plot shows six important protein parameters that will be used in protein predictive analysis in the future (Yallapragada et al, 2020).

PROFASA uses Canvas to create a radar plot of F2F Result, allowing users to intuitively see and compare the data of each protein, as shown in (Figure 5: *Ramachandran Plot and F2F Plot*). Two of the reasons for drawing manually rather than using an existing chart library, are the need for extensive flexibility and the need to keep the downloaded visuals consistent with what is displayed on the website. This is done with the Pixi.js library, which is the world’s fastest WebGL-2D rendering tool. In addition, PROFASA also has a document download feature for each protein sequence. Users can individually copy the sequence, download the PDB file, download the diagram, or download the complete PDF document. To display and download PDF files, the React PDF Renderer library is used.

PROFASA has always been built with a wide variety of users in mind, including professionals and non-professionals. As a result, the visual focus is stronger, the interaction mode is friendlier, and the learning costs are reduced.

### Back end design

The backend of PROFASA is written in JavaScript and runs in the Node.js environment, a runtime environment based on the Chrome V8 JavaScript Engine that runs outside the browser. For building Restful APIs, PROFASA uses KOA as a web server framework. KOA is a Node.js web framework with a powerful middleware system that can be used to handle request processing, CORS processing, token authentication, request interception, error handling, logging, and other operations elegantly. Furthermore, PROFASA uses Sequelize as an Object Relational Mapping (ORM) framework to build relationships between code data structures and database tables. Using Sequelize eliminates the need to write most of the SQL. It is very helpful for database migration and test environment setup. In version 2.0, the backend added the ability to take simple notes on the current model and the ability to share tasks between users. These features, although seemingly simple at the moment, are important changes in the long run when it comes to protein education and multi-person collaboration.

PROFASA uses Node.js not just to establish servers, listen to requests, and read and write databases, but also to run a variety of command-line scripts to perform complicated features. Open-source third-party programs and Python scripts produced by the developers themselves are examples of this. Command-line programs are run in the main Node.js program using the Node.js standard library’s child process.exec () and child process.execSync () methods. The most of these command-line programs are C or C++ compiled programs, or C or C++ programs launched by Python or the Shell. This provides these programs a lot of power and performance. In other words, PROFASA’s back end acts as a glue that holds together C or C++ programs that perform well and efficiently, but are difficult to install, difficult to use, and expensive to learn. For example, AlphaFold requires not only sophisticated hardware but also programming skills; The calculation process of I-TASSER is usually measured in days and cannot run continuously on PC; RpsBlast’s command-line program is almost unreadable to non-experts. Despite the complexity of the back-end logic, users can access the results of all these fantastic programs through the PROFASA front-end interface, which is simple to use and comprehend. Among them, the RpsBlast program does conservative domain queries, which is one of PROFASA’s fundamental features. RpsBlast searched according to Conserved Domain Database (CDD) and obtained a set of protein profiles (Camacho et al, 2008). However, in PROFASA, the RpsBproc program is used to parse and filter the RpsBlast results and perform ACD dataset searches against the results. The results are then parsed line by line, picking out useful information to display in the front end along with the RpsBproc results. As the most accurate and stable program for protein structure prediction in the non-neural learning area, the I-TASSER program is undoubtedly an important aspect of PROFASA. AlphaFold2, a tool developed by Google, is now the most advanced, powerful, accurate, and rapid neural learning protein structure prediction program on the market. Therefore, PROFASA added support for AlphaFold2.

The PROFASA server also supports calls to RCSB search, Protparam, isoelectric.org, and SAVES server, which are all well-known Protein APIs. PROFASA analyzes these API calls further and visualizes the intended outcomes, making it simple to learn about protein parameters and what they signify. As shown in (Table 2: *F2F plot parameters*), it lists the parameters in the F2F plot, some of which may be accessed using the APIs mentioned above. Among them, as shown in Formula 4, it can be used to calculate accessibility, which is defined as the relative accessible surface area (Marsh and Teichmann, 2011). Accessible Surface Area^5^ (ASA) is determined using the DSSP tool, whereas MaxASA is calculated by traversing all residues in PDB files and summing according to MaxASA for each residue, as shown in (Table 3: *MaxASA of amino acids*).

$$Accessibility=RSA=\frac{ASA}{MaxASA}=\frac{\sum ASA}{\underset{residue}{\sum }maxASA}$$ (4)

**TABLE 2:** F2F plot parameters.

F2F parameters	Scale range	Description	Generated
Size	kDa	Total weight of a protein	ProtParam Hosted by Expasy
RC.Score	0 to 100	Quality of a model	Saves Server
Accessibility	0 to 100	Active site solvent accessibility	PROFASA
Instability	0 to 100	Half-life of protein *in vitro*	ProtParam Hosted by Expasy
Hydrophobicity	−4.5 to +4.5	Surface hydrophobicity	ProtParam Hosted by Expasy
Isoelectric	0 to 14	Point at which molecule carries not net charge	Protein Isoelectric Point Calculator

**TABLE 3:** MaxASA^5^ of amino acids.

Residue	MaxASA	Residue	MaxASA
ALA	129.00	LEU	201.00
ARG	274.00	LYS	236.00
ASN	195.00	MET	224.00
ASP	193.00	PHE	240.00
CYS	167.00	PRO	159.00
GLU	223.00	SER	155.00
GLN	225.00	THR	172.00
GLY	104.00	TRP	285.00
HIS	224.00	TYR	263.00
ILE	197.00	VAL	174.00

Formula 4.

### Database design

**FIGURE 6:**
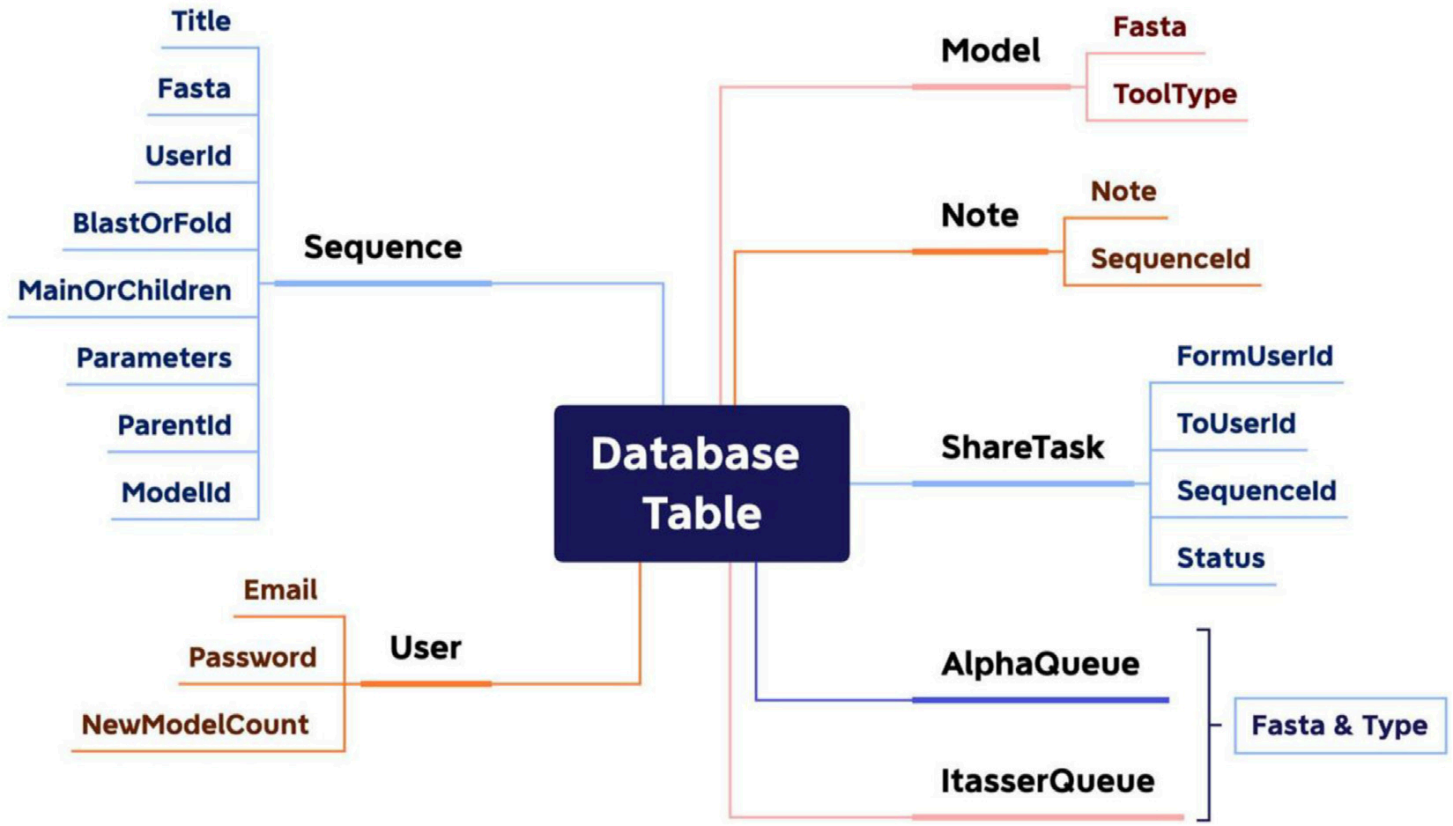
Database design.

Because PROFASA focuses on front-end interaction and back-end command line calls and results processing, the database was the easiest one of the project. All PROFASA database tables have three common fields: ID (Private Key), CreatedAt, and UpdatedAt. For the details, as shown in (Figure 6: *Database design*).

PROFASA: *Users can try PROFASA using this link:* https://profasa.ucc.ie/#/.

## Result

**FIGURE 7:**
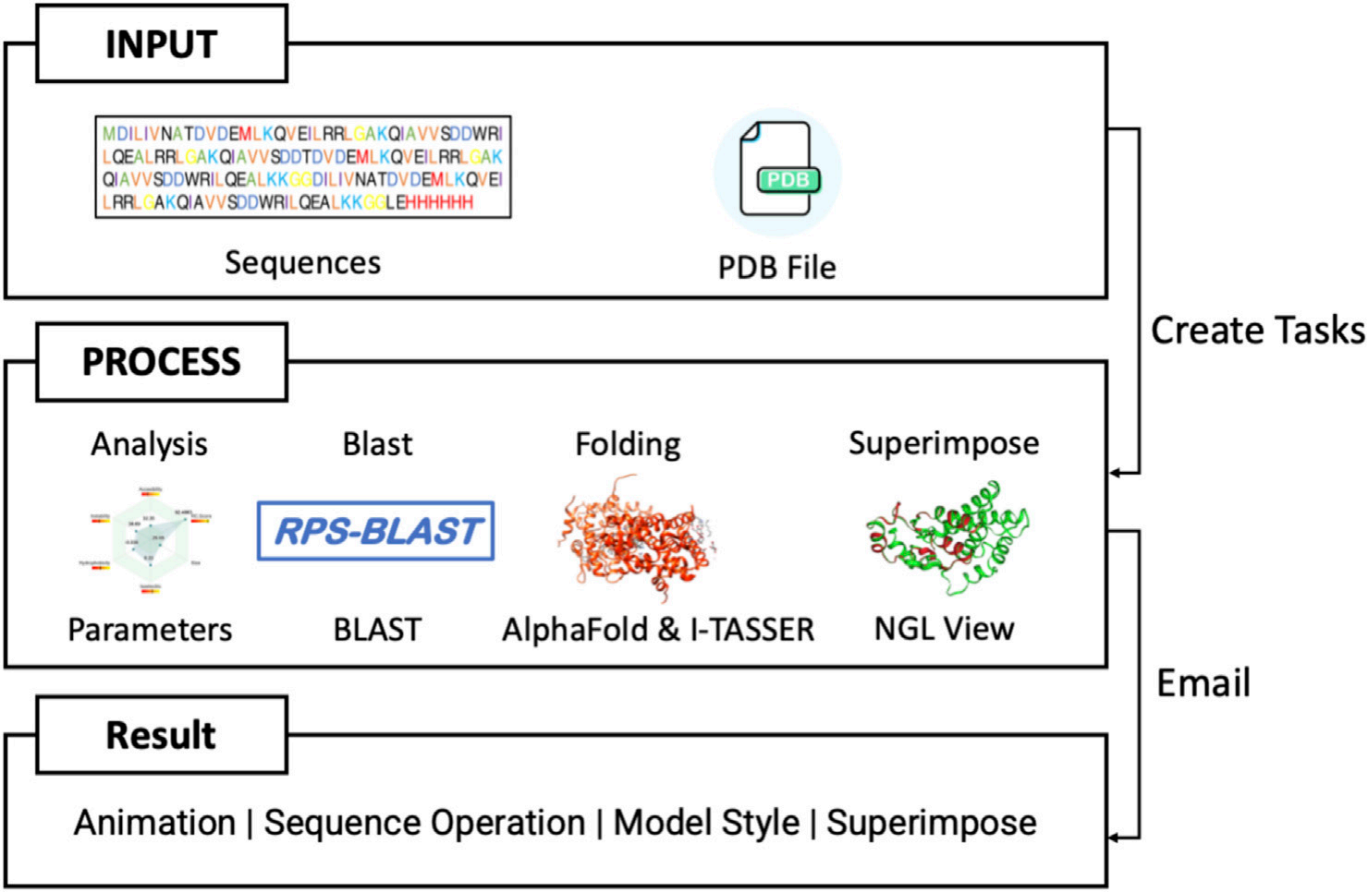
PROFASA flowchart.

PROFASA (Figure 7: *PROFASA flowchart*) is divided into four sections: Analysis, Superimpose, Structure Blast and the Fold. Each section takes a different input and provides outputs as discussed below.

### Analysis

**FIGURE 8:**
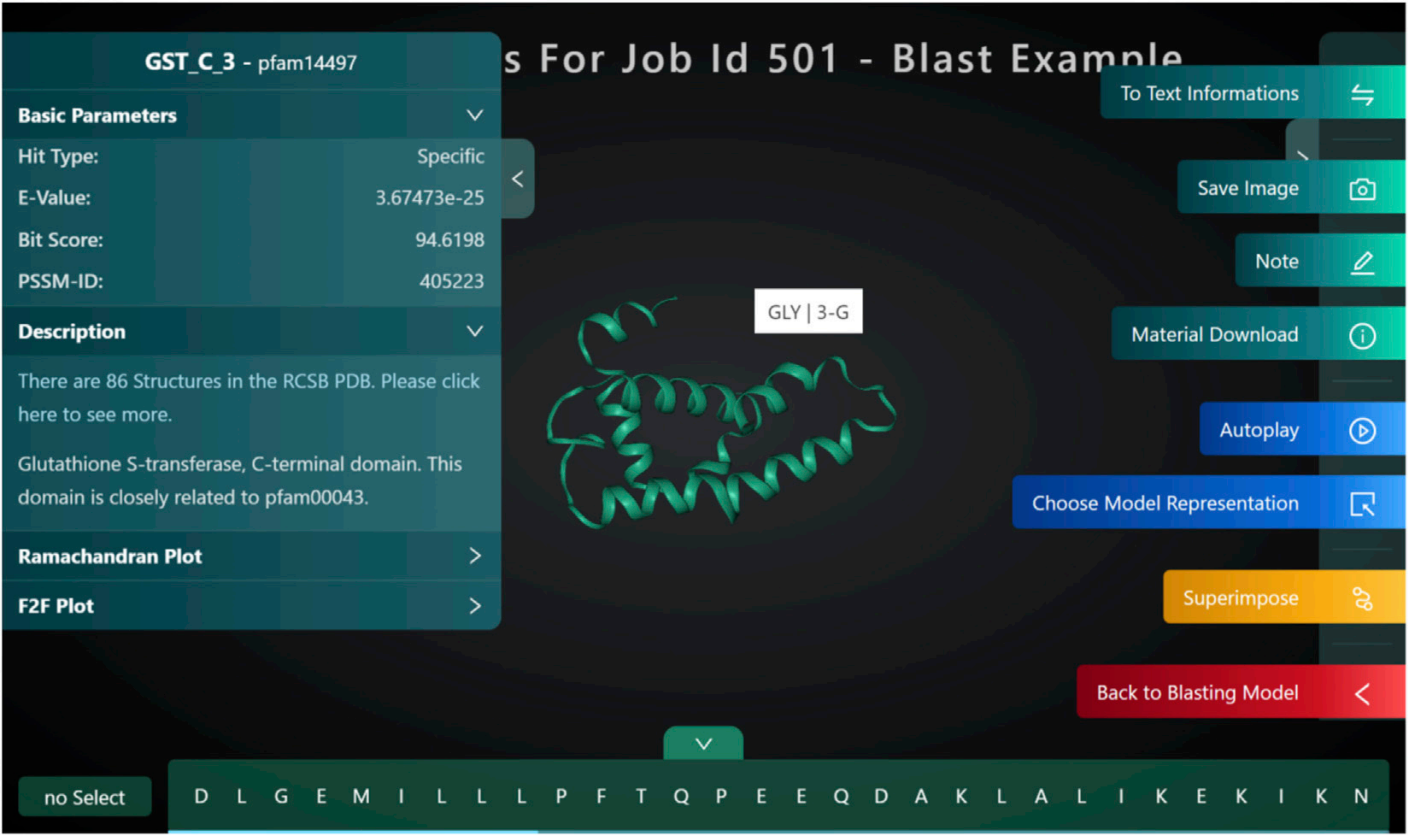
Analysis user interface.

To compute and understand various structural, sequence based and functional parameters of a protein (Figure 8: *Analysis user interface*), the user would upload a PDB file and PROFASA would compute all the parameters (as listed previously) and generate a PDF file.

### Superimpose

**FIGURE 9:**
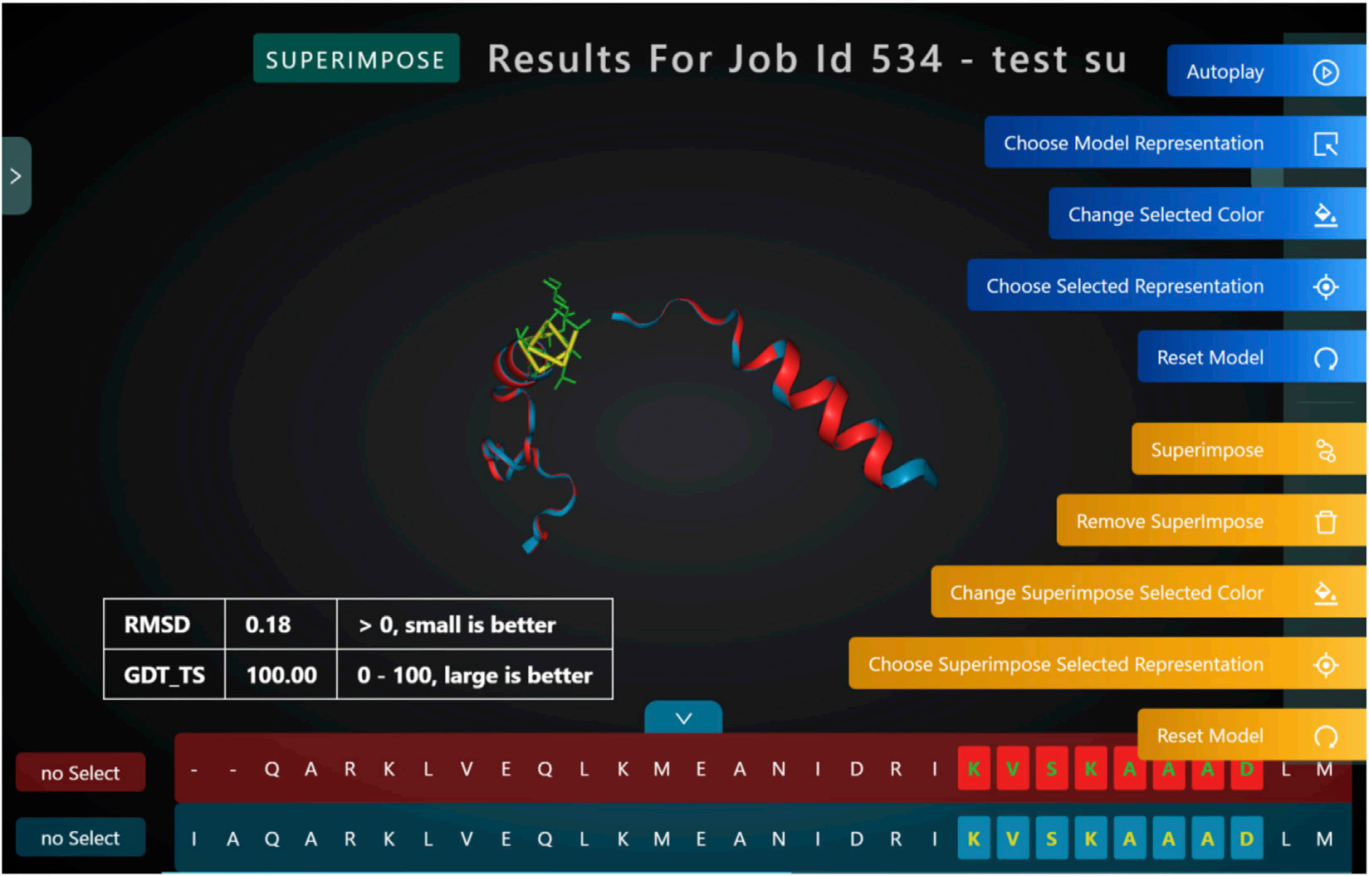
RMSD and GDT TS calculations are used to compare the differences between two superimposed 3D structures.

Superimposing two or more 3D structures is a powerful way to examine the differences in the structures. Biologists could use this for studying mutations, changes to functional hotspots, improving a novel fragment. PROFASA provides a 3D live image of the input PDB files, superimposed in the best configuration and calculates RMSD and GDT_TS to mathematically compare the difference between the structures (Figure 9: *RMSD and GDT TS*).

### Structure blast

**FIGURE 10:**
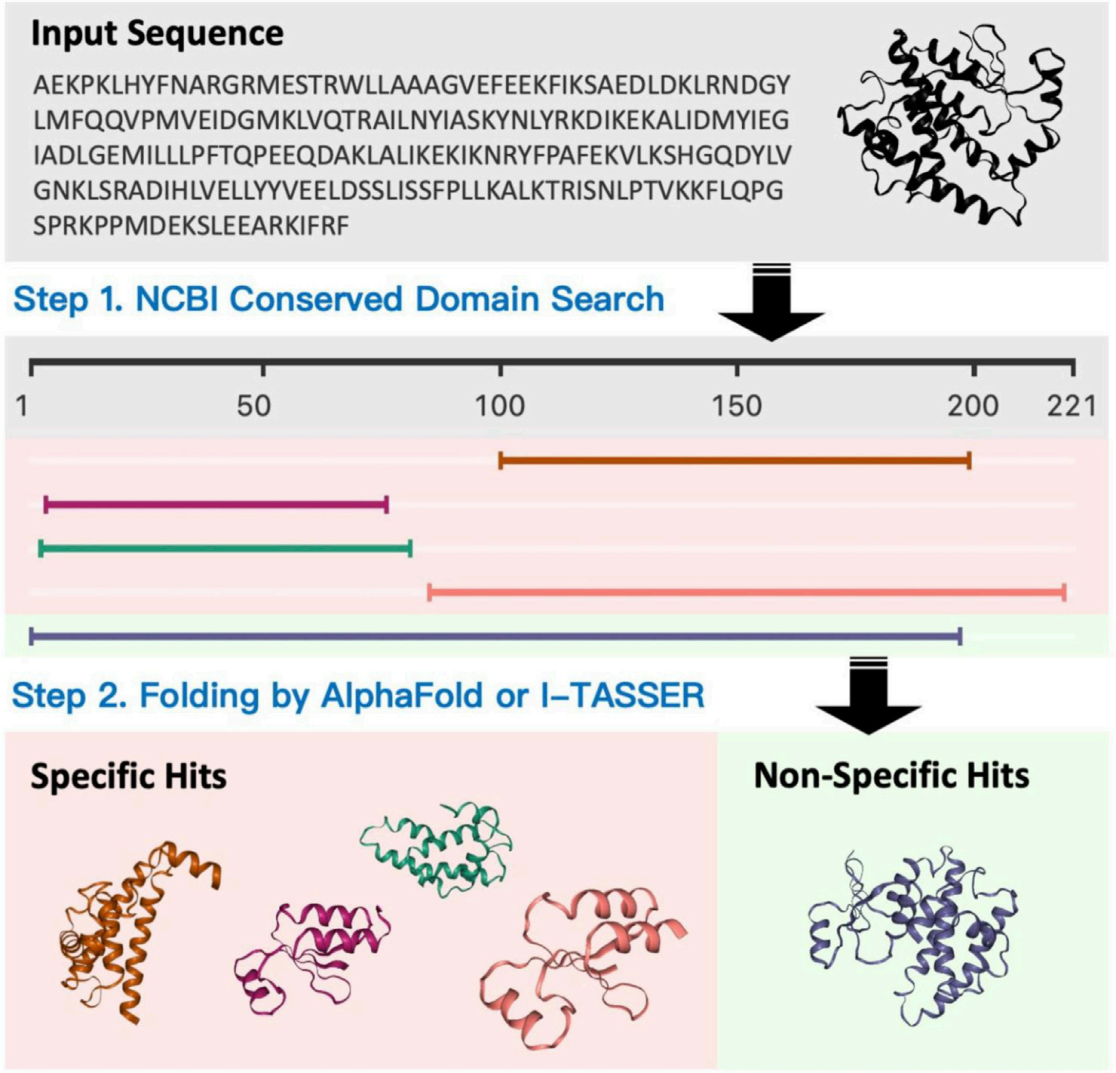
Structure blast.

Structure blast (Figure 10: *Structure blast*) is one of the unique features that we have developed for PROFASA. Traditional sequence based BLAST finds matches for the full and parts of a user defined sequence. PROFASA extends this a step further by giving a 3D model for the hits. These hits could either be modeled using I-TASSER or Alpha Fold for unknown sequences. As a result the user would be able to visualize how a large complex protein can have domains and parts from various smaller protein fragments existing in nature.

### Fold

PROFASA provides a user-friendly UI for protein folding or modeling. The user can fold multiple sequences in parallel and choose between I-TASSER and AlphaFold. The output is detailed in the in-built 3D visualisation tool along with all the computed parameters.

### Molecular visualisation

Having a clean, simple and interactive molecular visualisation embedded integrally into every result is a key for PROFASA’s highly user friendly interface. Unlike other protein analysis tools and folding software, users can access their 3D structure and interact with them on the same platform. Our web-based molecular visualisation is an important step that turns PROFASA into a one-stop-shop tool for biologists.

### Bio edutainment

The most important feature of PROFASA is the interaction with individual models, which sets it more characteristic than other protein analysis tools, as shown in (Figure 8: *Analysis user interface*). Hovering the mouse over the model reveals the amino acid at the present location as well as the sequence subscript. Additionally, the user could alter the color and style of the model area corresponding to a certain section of the sequence by selecting it by the sequence letter. Users also would be able to make notes on each structure and project. This level of interactivity makes PROFASA an excellent tool for teaching and bio edutainment.

## Discussion and outlook

### As a single UI protein workstation

**FIGURE 11:**
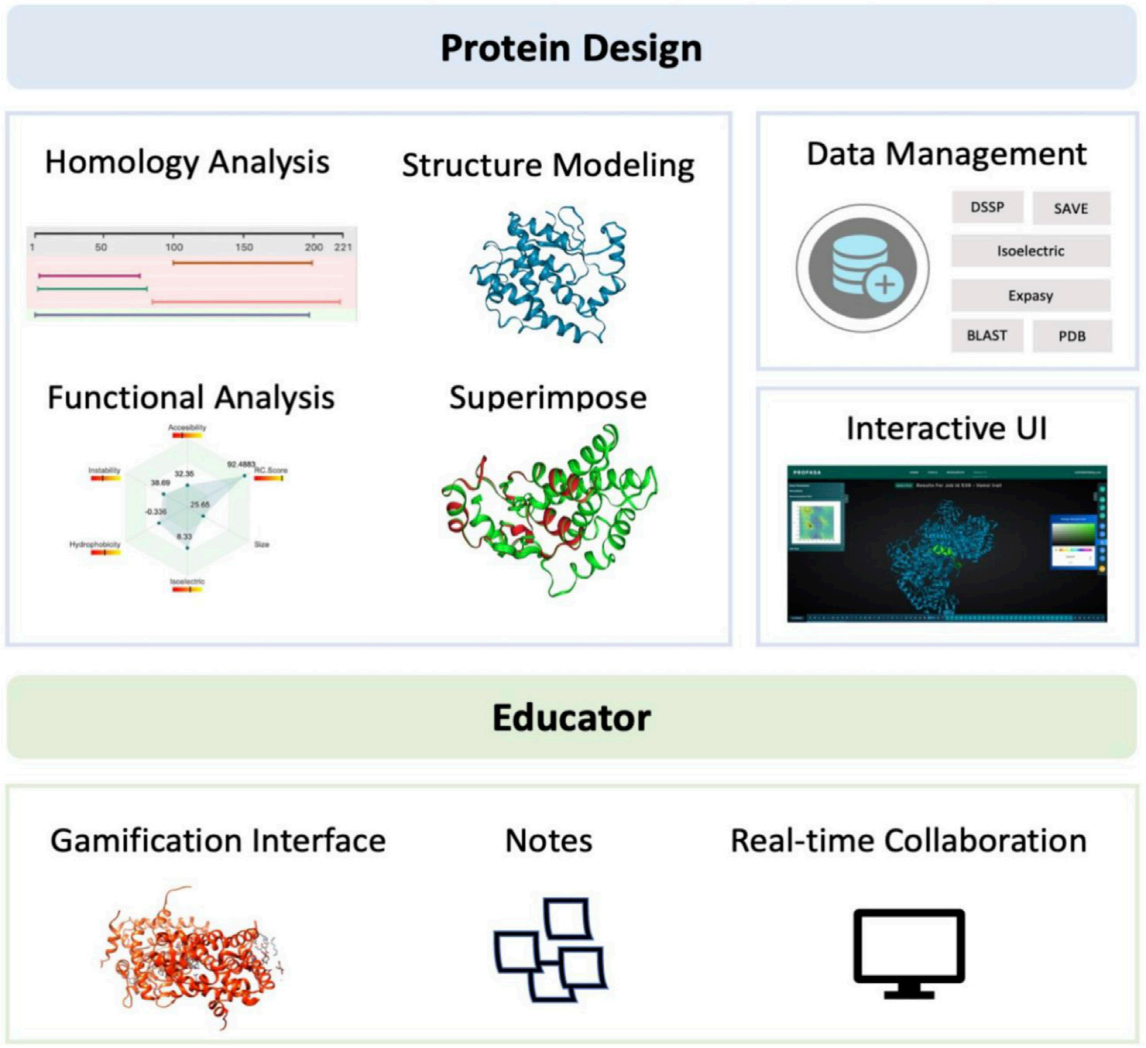
PROFASA functional classification.

Protein structure modeling and analysis is a crucial aspect in computational biology and bioinformatics, as it allows researchers to study the structure and function of proteins. Protein structures are complex, and determining their 3D structures experimentally can be time-consuming and expensive. Thus, computational methods are often used as a complement to experimental approaches. However, protein structure modeling and analysis are relatively difficult processes that frequently transition between many platforms or applications, posing a steep learning curve even for experienced computational biologists. Many of these tools and platforms require significant computational skills, as well as programming. This makes it challenging for biologists without a computational background to get started in the field. To address this problem, PROFASA, a one-stop protein structure modeling and analysis workstation, was created. The aim of PROFASA is to offer one platform for all protein sequence and structure analyses needs. It is worth noting that PROFASA has some important advantages over other existing tools, such as SAMSON Connect. For example, SAMSON Connect^6^ is an excellent integrated molecular design platform. Unlike the existing tools, PROFASA offers a one-stop solution with integrated tools (expanding library), robust data management, and no need for downloads as a web-based platform. Its user-friendly interface and low learning curve make it accessible to researchers without extensive computational backgrounds. We believe PROFASA is set to become an indispensable tool for researchers and educators in their quest to unravel the mysteries of protein structure and function. See Figure 11: *PROFASA functional classification*.

#### Protein design

PROFASA contributes significantly to the development of protein design as a one-stop workstation for modeling and analysis of protein structures. It combines into one platform many protein modeling and analysis tools such as, protein homology analysis, protein structure modeling, structure and function based parametric analysis, and protein structure superimposition. This enables researchers to quickly find potential ideas with a high chance of success without switching platforms. Another key advantage of the one-stop workstation is its ability to improve the accuracy and consistency of predictions by lowering the chance of practical mistakes and human error. PROFASA also enhances data management by enabling the integration and sharing of data obtained from multiple sources, facilitating the expansion of computational protein design. This can lead to the development of more sophisticated prediction techniques, and a large dataset can be accumulated to support the subsequent incorporation of machine learning algorithms. PROFASA has a user-friendly interface. This makes it easy to acquire and analyse protein structures for non-experts.

#### Educators

PROFASA was created with its use cases in the education field in mind from the start. It features powerful interactive molecular structure modification and gamified interface for molecular structure viewing, including high-definition 3D model display. Such a gamified user interface offers an immersive and interactive learning experience in contrast to traditional passive teaching techniques like lectures and textbook reading, and it has been shown to be a successful tool for science teaching and learning (Sailer and Homner, 2020). Further, PROFASA offers functionality for high-resolution note-taking on protein structures as well as peer-to-peer sharing of results from protein structure analyses. These features can meet the needs of both teachers who want to share teaching cases with students and students who want to submit learning outcomes to teachers. It meets the need for education to be enjoyable while simultaneously guaranteeing that instruction is effective and accurate. This makes it simpler to instruct students in computational biology, biochemistry, and biotechnology. Also, it helps to advance the training of researchers in the area.

### Bottlenecks and challenges for PROFASA

While PROFASA presents an innovative and efficient solution, one major challenge of PROFASA arises from the fact that PROFASA uses multiple external tools to perform its analyses, and the accuracy of the final results depends heavily on the accuracy of these external tools. Another challenge is that these analyses are computationally intensive processes, which can be both time-consuming and expensive to maintain. Additionally, given that the tool aims to generate large amounts of data, there is a need to ensure that the data is stored safely and securely, particularly if it is to be released openly. A further challenge is the need to continually improve the speed and accuracy of the tool, in order to keep pace with the rapidly evolving field of computational biology and bioinformatics.

### Ambitions and future outlook

**FIGURE 12:**
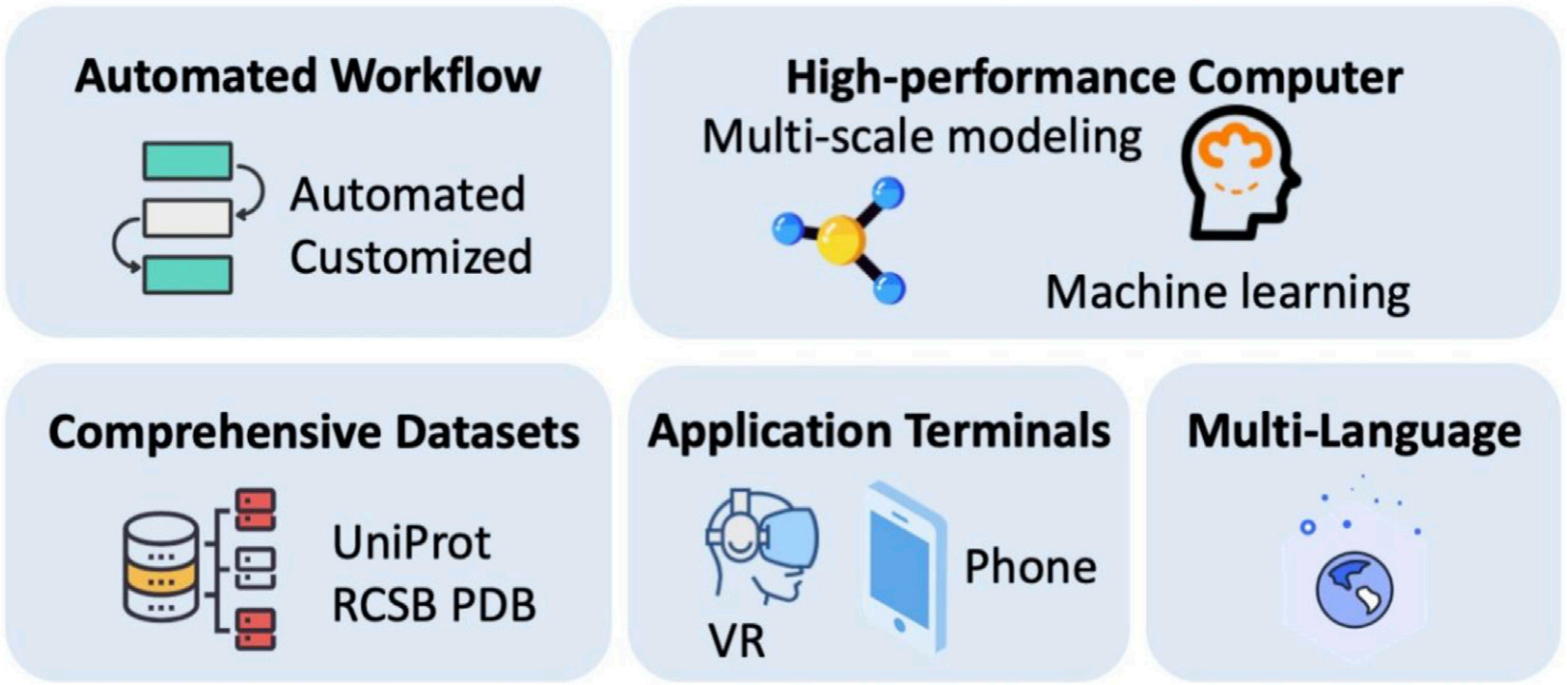
Ambitions and future outlook.

PROFASA already is a powerful tool for researchers and educators. PROFASA 2.0 is currently being created with more advanced features and ambitions, see Figure 12: *Ambitions and future outlook*. PROFASA 2.0 would include an automated workflow system to enhance the user experience for researchers with less experience and to expand data accessibility. Automation of parameter optimization, prediction validation, mistake detection, and report production are some of the features included in this automated workflow system. In addition to making protein structure modeling and analysis much simpler, this will also enable timely self-checking, which will lower the data error rate and raise the system’s degree of confidence.

Furthermore, PROFASA 2.0 would be upgraded with high-performance computational resources, like server clusters and load balancing and specialised GPU resources. This would allow capabilities for multi-scale modeling at various resolution levels, including coarse-grained, all-atomic, and explicit solvents. Additionally, PROFASA 2.0 will incorporate more comprehensive datasets to enhance protein prediction speed and accuracy in the future. These databases may include experimental data, functional annotations, information on protein evolution, and known protein structures and properties. PROFASA 2.0 would also be accessible through virtual reality headsets that brings an immersive learning experience to the users. Finally, the PROFASA 2.0 interface would also be available in multiple languages to extend its potential user base and make it more accessible to researchers from other locations.

## Data Availability

The original contributions presented in the study are included in the article/Supplementary material, further inquiries can be directed to the corresponding author.

## Footnotes

1
https://www.marketresearchfuture.com/reports/protein-engineering-market-691

2
https://predictioncenter.org/casp15/index.cgi

3
https://predictioncenter.org/casp14/doc/help.html

5
https://en.m.wikipedia.org/wiki/Relative_accessible_surface_area

6
https://www.samson-connect.net
